# Down-Regulated Drebrin Aggravates Cognitive Impairments in a Mouse Model of Alzheimer’s Disease

**DOI:** 10.3390/ijms18040800

**Published:** 2017-04-11

**Authors:** Yan Liu, Yanfeng Xu, Ling Zhang, Lan Huang, Pin Yu, Hua Zhu, Wei Deng, Chuan Qin

**Affiliations:** Comparative Medicine Center, Peking Union Medical College (PUMC) and Institute of Laboratory Animal Sciences, Chinese Academy of Medical Sciences (CAMS), Beijing 100021, China; sdcssm@sina.com (Y.L.); xuyanf2009@163.com (Y.X.); zhangling@cnilas.org.cn (L.Z.); huanglan0204@163.com (L.H.); pinyucau@gmail.com (P.Y.); zhuhua0226@vip.sina.com (H.Z.); dengwei717@163.com (W.D.)

**Keywords:** drebrin, Alzheimer’s disease, cognitive ability, hippocampus, APP/PS1 mice

## Abstract

The developmentally regulated brain protein drebrin (Dbn) is a functional protein involved with long-term memory formation and is widely distributed in brain neurons, especially in the dendritic spines. A noticeable decline of this protein has been found in the hippocampus and cortex of patients with Alzheimer’s disease (AD), yet the relationship between Dbn and AD has not been fully understood. In the present study, we examined how down-regulation of *Dbn* impacts the progression of AD in experimental animals. Accordingly, we injected *Dbn* interference vector (rAAV-m*Dbn1* ShRNA) into the hippocampus of three-month old APP(swe)/PS1(ΔE9) mice (APP/PS1 mice) and then successfully down-regulated *Dbn* expression in this brain region. Behavioral tests, including the Morris water maze test, the open field test, and the novel object test were conducted when the animals were nine months old. Subsequently, MicroPET/CT imaging to monitor glucose metabolism was done. We then investigated Aβ, GFAP, PSD-95, MAP2, vimentin, Cox43, and Syn1 expressions in the brain of the experimental animals via immunohistochemical or immunofluorescence methods. We found that AD mice with a low expression of Dbn performed poorly in the behavioral tests and showed decreased glucose utilization. In the brains of these animals, we detected a slight increase of Aβ, GFAP and vimentin and a significant decline of PSD-95. Altogether our data warrant further studies to elucidate the effect of Dbn on the development and progression of AD.

## 1. Introduction

The basic unit of information transmission between neurons is the synapse, which is composed of dendritic spines on the surface of dendrites and plays an important role in learning and memory [1,2]. The developmentally regulated brain protein drebrin (Dbn) contains adult type (Dbn A) and embryonic type (Dbn E) [3,4], distributed widely in the central nervous system and peripheral tissue. Dbn A (referred to as Dbn1 in mice) has been found in the function of maintaining long-term memory in vitro and is distributed in the neurons of the brain, especially in dendritic spines [5,6]. It has been found that Dbn is an F-actin binding protein, which conducts polymerization and depolymerization with F-actin to maintain or interrupt memory activities [7,8]. Studies report that *Dbn* depletion in wild-type mice reduced the dopamine receptor D1 (D1R), dopamine receptor D2 (D2R), 5-hydroxytryptamine receptor 1A (5-HT1AR), and 5-hydroxytryptamine receptor 7 (5-HT7R) protein levels in the hippocampus. Additionally, a noticeable reduction in memory-related hippocampal synaptic plasticity was found in electrophysiological studies of *Dbn* depleted mice. However, that was rarely reported in transgenic mice, and behavioral activities, cerebral glucose metabolism, or other indexes were rarely studied [2].

Alzheimer’s disease (AD) is a central nervous system degenerative disease with progressive development and is accompanied by learning and memory impairment [9]. Autopsies report that Dbn is decreased significantly in the hippocampus and cortex in AD patients [10,11]. There was a significant decrease in the hippocampus in AD model mice compared with the normal group [12]. This implies that Dbn may have a relationship with AD. However, it is not clear whether the loss of Dbn is caused by AD lesions or whether the reduction of Dbn accelerates the progression of AD. Whether down-regulated *Dbn* is the dominant factor in the change of cognitive ability and pathology in AD requires more detailed study.

The hippocampus is the main functional area of learning and memory activities and is more vulnerable to pathological changes after injury [13,14]. In addition, pathological changes of the hippocampus and cortex more often occurred in AD, including changes in morphology, protein level and distribution, hypometabolism, etc. [15,16]. Among these, alterations of the hippocampus and in cognition are often used to evaluate the effect of research into and treatment of AD [17,18]. In-depth research on the effects of down-regulated *Dbn* in AD model animals on behavior, metabolism, or molecular level is rarely reported, and the hippocampus is more prone to lesions in AD. We injected rAAV9-m*Dbn1* ShRNA vectors into the hippocampus of APP/PS1 transgenic mice to maintain a low-level expression of Dbn for months. The behavioral performances, glucose metabolism, and related proteins in mice were investigated to explore the impact of decreased Dbn on AD and provide experimental cues for future research and treatment of AD.

## 2. Results

### 2.1. RAAV9-mDbn1 ShRNA Vector Produced and Injected into the Hippocampus of AD Mice 

**Figure 1:**
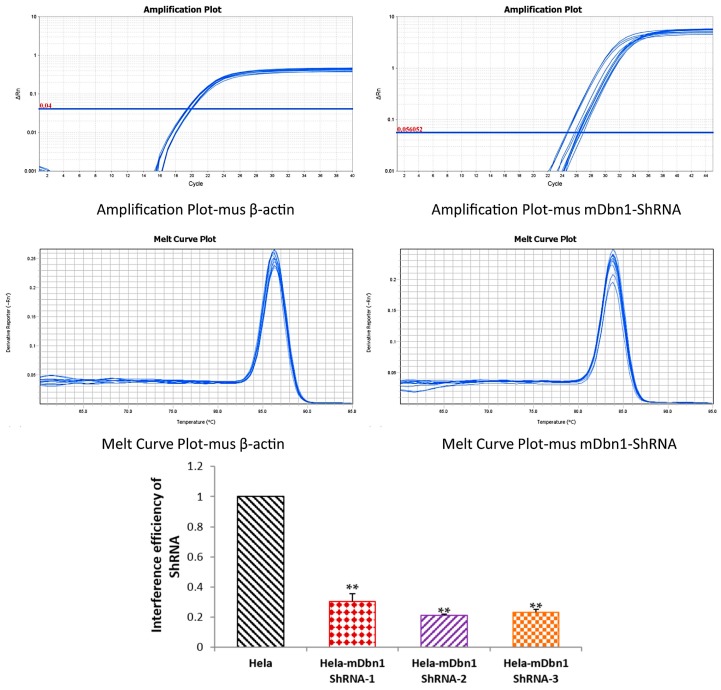
Interference efficiency of ShRNA-*Dbn1* in Hela cell was detected by Q-PCR (* *p* < 0.05, ** *p* < 0.01 vs. Hela).

According to m*Dbn1* (NM_001177371) gene sequences, rAAV9-ZsGreen-m*Dbn1* ShRNA vectors were produced and purified. Three candidate ShRNA sequences were designed based on the m*Dbn1* coding region (Table 1). The plasmid vectors pAAV-ZsGreen-ShRNA containing BamHI+HindIII (Figure S1) were connected with the three candidate sequences. Sequence comparison shows complete consistency between pAAV-ZsGreen-m*Dbn1* ShRNA and the design sequence. The interference efficiency of the vectors in Hela recipient cells was evaluated by real-time Q-PCR test (Figure 1), and then the most efficient ShRNA vector (Sequence 2) was selected and produced to be a rAAV9-m*Dbn1* ShRNA vector with a high titer 5 × 10^12^ vg/mL. The hippocampus of three month old AD mice was injected with 2.5 μL rAAV-zsGreen-Sh*Dbn1* vector per side to form the APP/PS1-ShDbn1 group (bilateral injection). Similarly, volume-matched rAAV-zsGreen vector was injected into age-matched AD mice as APP/PS1-control mice. All mice were reared until nearly nine months of age. Behavioral tests and MicroPET/CT inspection were conducted, and then the mouse brains were extracted for the following experiment.

### 2.2. Cognition Changes in Mice

#### 2.2.1. Morris Water Maze Test

**Figure 2:**
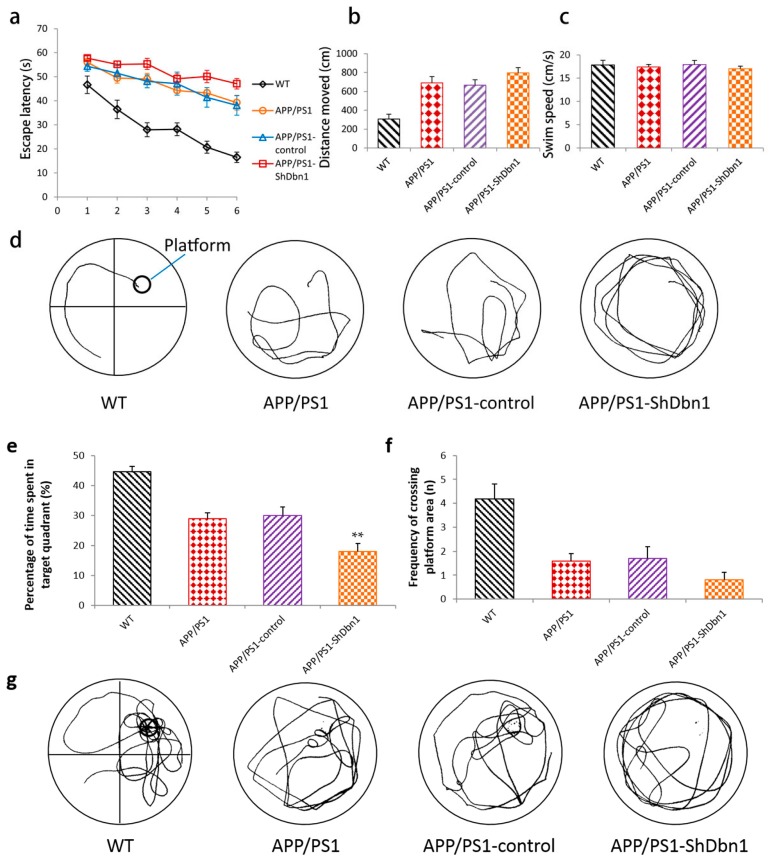
Morris water maze test (MWM) performance of mice in different groups (WT, APP/PS1, APP/PS1-control, APP/PS1-ShDbn1). (**a**) Escape latency; (**b**) distance moved; (**c**) swim speed; (**d**) swim traces in training test; (**e**) percentage of time spent in target quadrant; (**f**) frequency of crossing platform area; and (**g**) swim traces in probe test (*n* = 11 for WT, *n* = 10 for APP/PS1, *n* = 10 for APP/PS1-control, *n* = 11 for APP/PS1-ShDbn1, ** *p* < 0.01 vs. APP/PS1-control).

The Morris water maze test (MWM) is always used to investigate the ability of learning and memory in AD mice. The results of MWM are depicted in Figure 2. In the hidden platform test stage, the escape latency in all groups was shortened with the increase of training days. However, the time to board the platform in AD and APP/PS1-ShDbn1 mice was significantly longer than Wild type(WT) mice beginning from the second day of training (*p* < 0.01, Figure 2a). Though there were no noticeable differences between the APP/PS1-control and APP/PS1-ShDbn1 groups in escape latency and swim speed (*p* > 0.05, Figure 2a,c), the APP/PS1-ShDbn1 mice required more time to board the platform beginning from the third day (Figure 2a,d). The distance moved in the APP/PS1-ShDbn1 mice (796.43 ± 54.98) was longer than that of the APP/PS1-control mice (665.67 ± 57.29, Figure 2b). In the probe test stage, the percentage of time spent in the target quadrant of APP/PS1-ShDbn1 mice was markedly less than APP/PS1-control mice (*p* < 0.01, Figure 2e). Though there were no significant differences between the APP/PS1-control and APP/PS1-ShDbn1 groups in the frequency of crossing the platform area, the frequency in APP/PS1-ShDbn1 mice (0.82 ± 0.30) was less than that of the APP/PS1-control mice (1.7 ± 0.50, Figure 2f). The results show that the learning and memory ability in APP/PS1-ShDbn1 mice decreased after injection.

#### 2.2.2. Open Field Test

**Figure 3:**
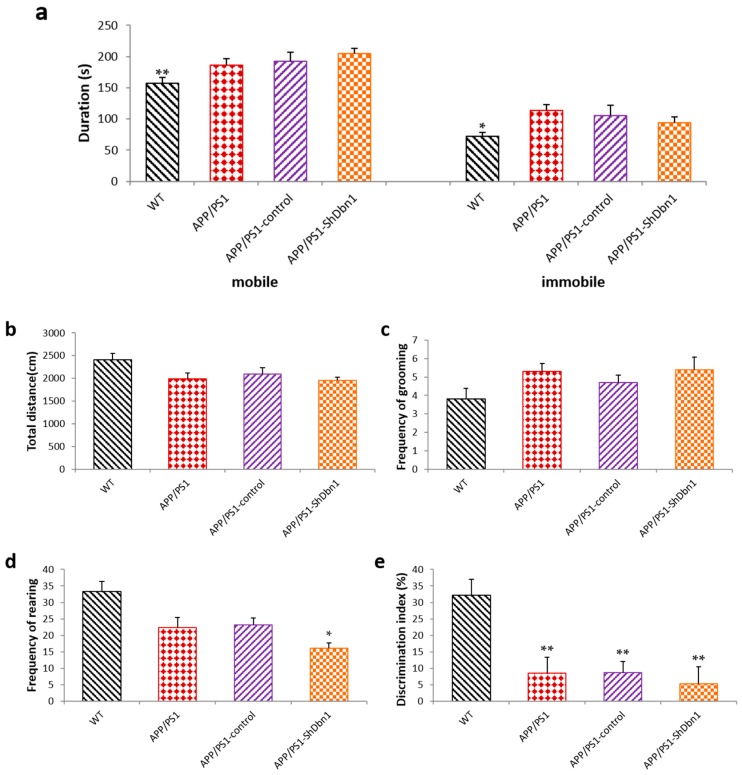
Results of open field test and novel object test (**a**) Frequency of mobile and immobile (* *p* < 0.05, ** *p* < 0.01, APP/PS1-ShDbn1 vs. WT); (**b**) total distance; (**c**) frequency of grooming; and (**d**) frequency of rearing in open field test (*n* = 11 for WT, *n* = 10 for APP/PS1, *n* = 10 for APP/PS1-control, *n* = 10 for APP/PS1-ShDbn1, * *p* < 0.05, ** *p* < 0.01 vs. APP/PS1-control); (**e**) DI in novel object test (*n* = 11 for WT, *n* = 10 for APP/PS1, *n* = 9 for APP/PS1-control, *n* = 10 for APP/PS1-ShDbn1, * *p* < 0.05, ** *p* < 0.01 vs. WT).

An open field test was conducted after the MWM test to explore the spontaneous motor activities and anxious mood in mice. The result shows that the duration of mobile (*p* < 0.01) and immobile (*p* < 0.05) activity of APP/PS1-ShDbn1 mice was significantly increased compared with the WT mice (Figure 3a). This implies that the APP/PS1-ShDbn1 mice appeared to be markedly anxious compared with the WT mice. There were no significant differences in total distance and frequency of grooming (*p* > 0.05, Figure 3b,c), but the frequency of rearing in APP/PS1-ShDbn1 mice was markedly less than that of the APP/PS1-control mice (*p* < 0.05, Figure 3d). These results imply that the spontaneous motor activities of the APP/PS1-ShDbn1 mice were reduced and anxious mood increased after injection.

#### 2.2.3. Novel Object Test

In the novel object test, the Discrimination Index (DI) in all AD groups is noticeably lower than that of WT mice (*p* < 0.01, Figure 3e). This indicates that the ability to explore novel objects is markedly decreased in AD mice. The DI of APP/PS1-ShDbn1 mice (5.27 ± 5.14) is lower than that of APP/PS1-control mice (8.67 ± 3.37, *p* > 0.05), although no significant differences are found in Figure 3e.

### 2.3. Glucose Metabolism Changes in Mice 

**Figure 4:**
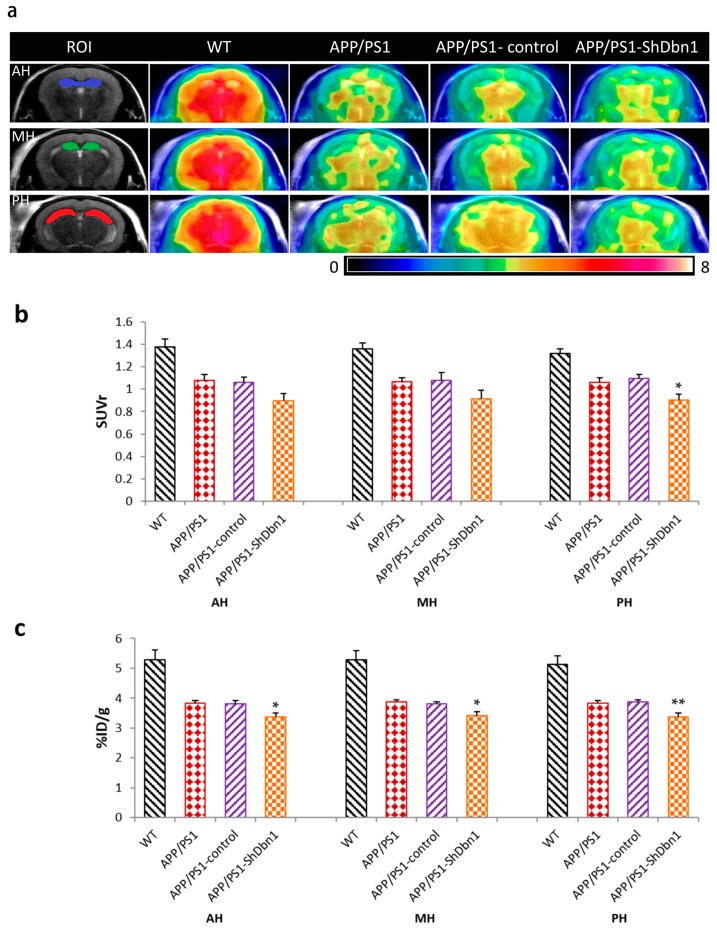
Results of MicroPET/CT. (**a**) Regions of interest (ROI) and PET/CT images in all groups mice (*n* = 5 per group); (**b**) relative standardized uptake value (SUVr) and % injected dose per gram of ^18^F-FDG (%ID/g) in ROI (* *p* < 0.05, ** *p* < 0.01 vs. APP/PS1-control).

To further study the impact of rAAV-*Dbn1* ShRNA in AD hippocampus, the glucose metabolism in mice was detected by MicroPET/CT. We selected anterior, middle, and posterior regions of the hippocampus (AH, MH, PH) as regions of interest (ROI, Figure 4a) to evaluate relative standardized uptake values (SUVr) and the percentage of injected dose per gram of ^18^F-FDG (% ID/g). Compared with the APP/PS1-control mice, the SUVr of the PH region in the APP/PS1-ShDbn1 group was noticeably reduced (*p* < 0.05, Figure 4b). The % ID/g of APP/PS1-ShDbn1 mice was significantly lower than that of APP/PS1-control mice (*p* < 0.05 in AH and MH regions, *p* < 0.01 in PH region, Figure 4c). These results suggest that glucose utilization in APP/PS1-ShDbn1 within the hippocampus was reduced markedly compared with the no ShRNA injection AD mice.

### 2.4. Dbn Protein Significantly Decreased in RAAV9-ShDbn1 Vector Injection Area of AD Mice

**Figure 5:**
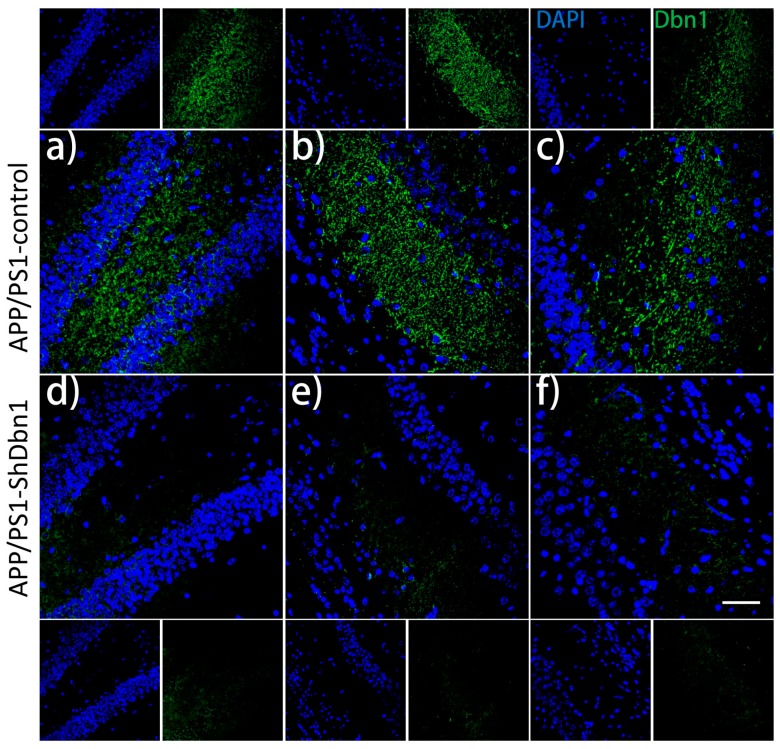
Immunofluorescence staining of Dbn in the rAAV-ShRNA injection group and without ShRNA APP/PS1 mice. (**a**–**c**) and (**d**–**f**) show different regions (DG, CA2, and CA3) of the hippocampus (*n* = 7 per group, Scale bar = 50 µm).

**Figure 6:**
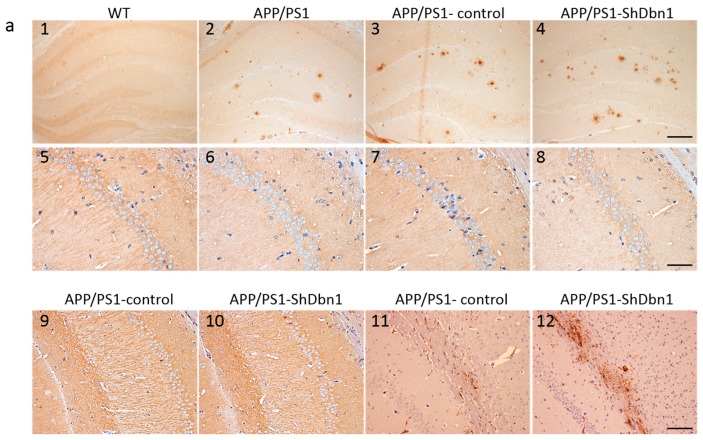

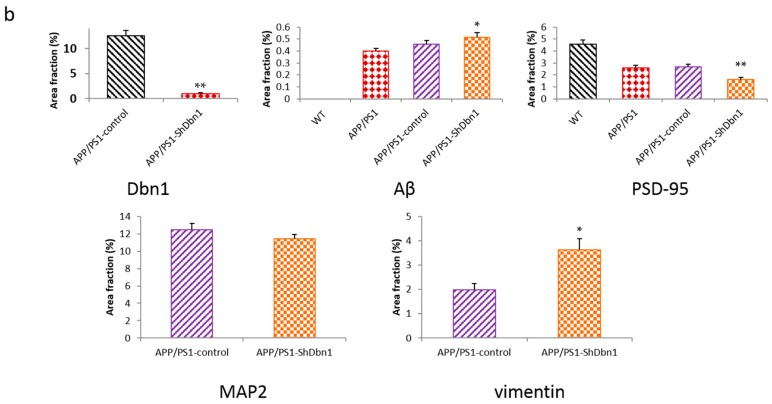
Results of immunohistochemical staining (**a**) amyloid β plaques (**1**–**4**), PSD-95 protein (**5**–**8**), MAP2 (**9**,**10**), and vimentin (**11**,**12**) expression in the hippocampus (*n* = 6 per group, scale bar: (**1**–**4**), 200 μm; (**5**–**8**), 40 μm; (**9**–**12**), 100 μm); (**b**) Area fraction of Dbn1, 4G8 and PSD-95 in mice (* *p* < 0.05, ** *p* < 0.01 vs. APP/PS1-control).

To verify the interference effect of rAAV-ShRNA, Dbn was detected by immunofluorescence staining in the hippocampus of the rAAV-ShRNA injection group and without ShRNA injection AD mice, and then images in different regions (DG, CA2, and CA3) were captured. The results showed that the expression level of Dbn was markedly reduced in APP/PS1-ShDbn1 mice compared with AD mice (*p* < 0.01, Figure 5 and Figure 6b). These results demonstrate that Dbn expression in the hippocampus of APP/PS1-ShDbn1 mice was efficiently down-regulated by rAAV-ShRNA.

### 2.5. Aβ and GFAP Increased Within a Small Scale in Dbn1 Down-Regulated AD Mice

**Figure 7:**
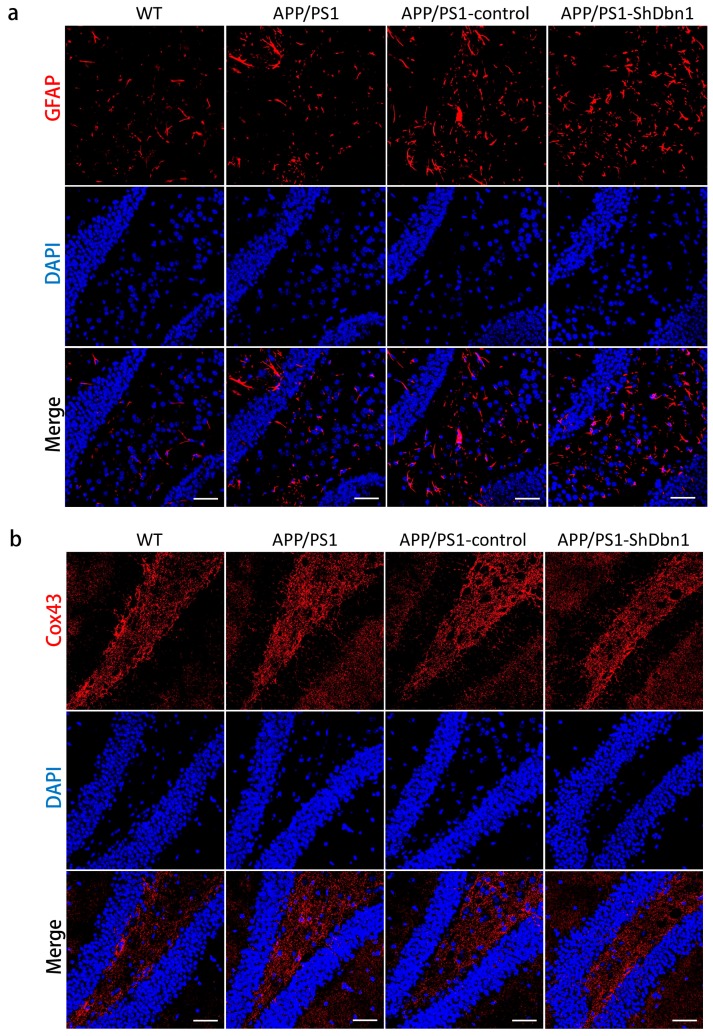
Immunofluorescence staining of GFAP and Cox43 in the hippocampus. (**a**) GFAP expression in the hippocampus; (**b**) Cox43 expression in the hippocampus (*n* = 6 per group, scale bar = 50 μm).

**Figure 8:**
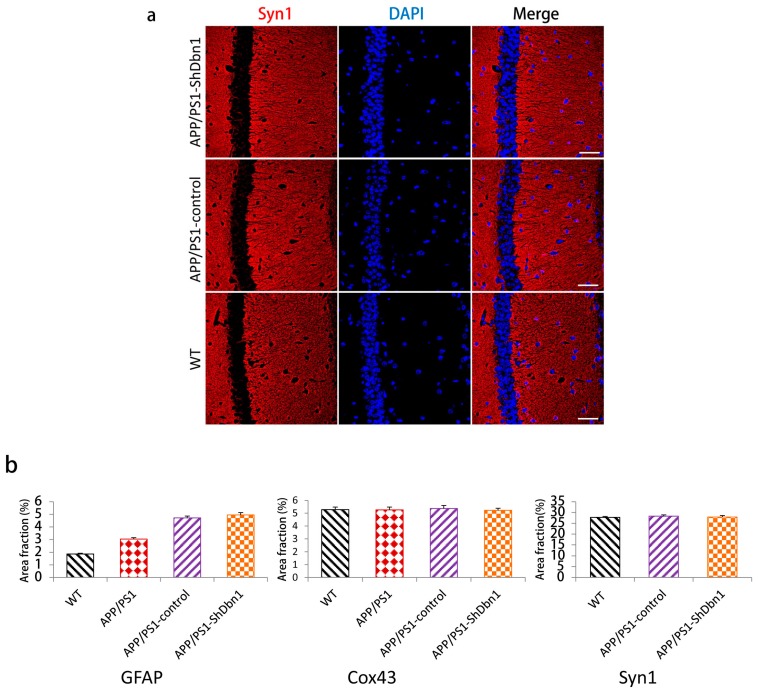
Results of immunofluorescence staining. (**a**) Expression results of Syn1 (scale bar = 50 μm); (**b**) area fraction of GFAP, Cox43, and Syn1 (*n* = 6 per group).

To explore the impact of down-regulated *Dbn1* in AD mice, Aβ and GFAP in the hippocampus were detected by immunohistochemical or immunofluorescence staining (Figure 6a and Figure 7a). Aβ plaque was a typical pathological change in the AD brain. In addition, GFAP was a specific marker of astrocytes to assess the homeostasis, inflammation, and nutrition of the brain [19,20,21]. The area fraction data showed that Aβ and GFAP expression in APP/PS1-ShDbn1 was higher than in APP/PS1-control mice, though no statistically significant difference was found (Figure 6b and Figure 8b). GFAP in injection mice (APP/PS1-control and APP/PS1-ShDbn1) was significantly greater than AD mice without injection (*p* < 0.01, Figure 8b). The area fraction of Aβ in APP/PS1-ShDbn1 mice (0.52 ± 0.04) was more than that of the APP/PS1-control group (0.46 ± 0.03, *p* = 0.24, *p* > 0.05, Figure 6b). In addition, the area fraction of GFAP in the APP/PS1-ShDbn1 (4.95 ± 0.18) mice was higher than the in the APP/PS1-control group (4.72 ± 0.15, *p* = 0.33, *p* > 0.05, Figure 8b). The data imply that a lack of Dbn may promote production of Aβ and activation of astrocytes and then aggravate lesions associated with AD.

### 2.6. Expression Level of Relative Protein in Mice Hippocampus

For further study of changes in Dbn relative protein in mice hippocampus, postsynaptic density-95 (PSD-95), microtubule-associated protein 2 (MAP2), vimentin, connexin43 (Cox43), and synapsin1 (Syn1) were detected by immunohistochemical or immunofluorescence methods. PSD95 is a marked protein, which can combine with Dbn to maintain memory activity in neuronal dendritic spines [8]. Compared with WT mice, the area fraction of PSD-95 in all AD mice was significantly decreased. PSD-95 in the APP/PS1-ShDbn1 mice was noticeably lower than in APP/PS1-control mice (*p* < 0.01, Figure 6a,b). The results imply that lack of Dbn may impact PSD-95 expression in the hippocampus. MAP2, a marker protein, was found specifically in dendritic branches of the neuron. Though there was no significant difference between the APP/PS1-control and the APP/PS1-ShDbn1 mice, the area fraction of MAP2 in APP/PS1-control mice (12.46 ± 0.73) was higher than that in APP/PS1-ShDbn1 mice (11.41 ± 0.49). Vimentin, an intermediate filament (IF) protein, plays important roles in cytoskeletal constitution and cell signal transduction [22,23]. In this study, vimentin was found in the hippocampus of APP/PS1-ShDbn1 mice markedly more than in APP/PS1-control mice (*p* < 0.05, Figure 6a). This implies that down-regulated *Dbn* may impact vimentin greatly. Cox43, a highly expressed connexin widely spread throughout brain tissue, can interact with Dbn [24,25,26]. Synaptic associated protein Syn1 performed various functions in synapse formation, vesicle trafficking, and neurotransmitter release and was found decreased in the AD brain [27]. This study investigated the expression level of the two proteins by immunofluorescence staining. However, there were no significant differences in Cox43 and Syn1 levels between the APP/PS1-ShDbn1 and APP/PS1-control mice (*p* > 0.05, Figure 7b and Figure 8). The data imply that decreased Dbn has no noticeable impact in these two proteins.

## 3. Discussion

To investigate the role of Dbn in AD mice hippocampus, rAAV9-m*Dbn1* ShRNA vector was injected into the hippocampus of AD mice. This study successfully down-regulated the expression of Dbn in the target region. Behavioral tests to assess cognition abilities and MicroPET/CT to evaluate glucose metabolism changes were conducted when the mice were nine months old. A MWM test was conducted to judge the learning and memory capabilities of the rodents [28]. In MWM training, the APP/PS1-ShDbn1 mice performed poorly in search platform and probe target quadrant tests. An open field test was used to investigate autonomous activities and anxiety [29]. The results found reduced autonomous activities and increased anxious mood in the APP/PS1-ShDbn1 mice. Researchers have found a marked decline in glucose uptake by PET/CT inspection in the hippocampus and cortex of AD patients [30,31]. That is, this demonstrates that degenerative disease in the central nervous system may have a glucose metabolism disorder. MicroPET/CT was performed in mice to explore the impact of glucose utilization of Dbn to AD mice. We found a significant decline in glucose utilization in lacked-Dbn AD mice compared with AD mice in the hippocampal region. Increased anxiety, impaired cognitive capability, and glucose utilization in APP/PS1 mice were also found in other studies [32,33,34]. Glucose utilization is a main pathway to provide cerebral energy and maintain blood glucose [35]. The results show that APP/PS1-ShDbn1 mice have a low glucose metabolism condition in the hippocampus, which may be one factor influencing behavioral performances.

Aβ plaque is a typical pathological marker in the AD brain that is used to evaluate the progression of AD and can interact with astrocytes [36]. Increased Aβ may activate astrocytes; conversely, activated astrocytes can promote Aβ increase by the secretion of inflammatory factors [37,38]. In addition, Aβ toxicity and neuroinflammation can accelerate synaptic impairment and neuron death in AD patients [39,40]. GFAP is a specific marker to detect astrocytes, which can directly reflect morphology and the quantity of astrocytes and then estimate cerebral status. We detected Aβ plaque and GFAP by immunohistochemical and immunofluorescence methods. In this study, there were slight scale increases of Aβ and GFAP in the APP/PS1-ShDbn1 mice compared with the APP/PS1-control mice and the APP/PS1 mice. The data suggests that lack of Dbn may promote production of Aβ and the activation of astrocytes and then aggravate lesions of AD.

Postsynaptic density protein PSD-95 is a neuron marker used to evaluate synaptic morphological and functional changes. It was found to be significantly decreased in the AD brain [41,42]. PSD-95 was investigated through immunohistochemical staining in the hippocampus of mice. Compared with APP/PS1-control mice, the area fraction of PSD-95 and MAP2 in APP/PS1-ShDbn1 mice was reduced. This may provide evidence that down-regulated *Dbn* promotes PSD-95 and MAP2 decrease in AD. Diabetes is a risk factor of AD, and AD is more prone to glucose metabolism disorders [43]. Studies showed Dbn down-regulated by single-minded 2 (Sim2) resulted in diabetic glucose utilization disorder and memory impairment in rats [44]. Dbn, which is a key protein in memory activities, can stabilize PSD-95 at synapses and then perform the communication between neurons. The neuronal activities are provided with energy and nutrition by astrocytes. Down-regulated Dbn and PSD-95 block effective neuronal activities and aggravate impairment of AD. As mentioned previously, sedimentary Aβ plaque can promote abnormal astrocyte activation, which may lead to glucose utilization disorder. Astrocytes have another function in the inhibition of adult neurogenesis depending on increased vimentin. Microglias are also immune cells that produce high levels of pro-inflammatory factors, causing neurotoxicity. Vimentin can activate microglia and astrocytes to aggravate neurotoxicity and impairment. Deleted vimentin can alleviate microglia activation and neuron damage [45,46]. In this study, a high-level of vimentin increase was observed in the APP/PS1-ShDbn1 mice. This is one possible one factor leading to aggravated AD impairment. For further study, connexin Cox43 and synaptic associated protein Syn1 were detected by immunofluorescence staining. Cox43 is widely spread in brain tissue and can interact with Dbn. Syn1 is an important synaptic marker in the AD brain. However, there was no significant difference found between APP/PS1-ShDbn1 and APP/PS1-control mice. Though this research has some limitations, it still provides some behavioral, glucose metabolism and molecular detection resources for down-regulated *Dbn* in AD that were rarely reported before. Dbn is a key protein, which was down-regulated in the hippocampus and can aggravate the impairment of Alzheimer’s disease in many aspects. This study may provide a target protein to assist in clinical treatment. 

## 4. Materials and Methods

### 4.1. Animals

APPswe/PSΔE9 (APP/PSI) transgenic Alzheimer’s disease model mice (C57BL/6J background) and Wild type C57BL/6J mice (WT) were provided by the Institute of Experimental Animals of the Chinese Academy of Medical Science. All animals were maintained at 22–23 °C and at a 12-h light/dark cycle with ad libitum water and food. All animal studies were approved by the Institutional Animal Care and Use Committee of the Institute of Laboratory Animal Science of Peking Union Medical College (QC16002, Date of approval: 6 May 2016). All mice of the same sex (female) and age were divided into four groups for experiments. Due to female APP/PS1 mice being more prone to cognitive and pathological damage, 11 female AD mice were injected with rAAV-zsGreen-Sh*Dbn1* vector at three months as the APP/PS1-ShDbn1 group. 10 age-matched AD mice were injected with rAAV-zsGreen vector as the APP/PS1-control group. 10 sex and age matched APP/PS1 mice and 11 sex and age matched C57BL/6J mice without any injection formed the APP/PS1 and WT groups. All mice performed behavioral tests and underwent MicroPET/CT inspection six months after injection, and brain tissue was extracted for the following molecular experiments. 

### 4.2. RAAV9-mDbn1 ShRNA Vector Production and Purification

The rAAV9-ZsGreen-m*Dbn1* ShRNA vectors were produced and purified according to m*Dbn1* (NM_001177371) gene fragment standards by Viraltherapy technologies (Wuhan, China). Three ShRNA sequences were designed based on the m*Dbn1* coding region (Table 1). The plasmid vectors pAAV-ZsGreen-ShRNA containing BamHI + HindIII were respectively connected with the three sequences and investigated through sequence analysis. Sequence comparison shows complete consistency between pAAV -ZsGreen-m*Dbn1* ShRNA and the design sequence. To select the most efficient ShRNA vector of the three, the interference efficiencies of the vectors in Hela recipient cells were assessed by real-time Q-PCR test (Figure 1). The candidate vector (Sequence 2) was produced to rAAV9-m*Dbn1* ShRNA vectors. Subsequently, the vectors were purified with a high titer 5 × 10^12^ vg/mL.

### 4.3. Intrahippocampal Injection

We used the mouse brain stereotaxic apparatus to perform intrahippocampal injection. Mice were anaesthetized and secured with an ear bar in prone position. The skin was sterilized and cut. According to the book, “The Mouse Brain in Stereotaxic Coordinates” [47], an accurate injection point, which is marked with a marker on the skull, is bregma posterior 2.1 mm, left or right side, to the sagittal suture 1.35 mm. The injection depth was measured by the dividing rule with 1.75 mm during the injection process. Holes were drilled in the skull according to the injection point with a dental drill. 2.5 µL rAAV-zsGreen-Sh*Dbn1* or rAAV-zsGreen vector with a titer 5 × 10^12^ vg/mL was injected into AD mice hippocampi on each side with a 10 μL capacity microinjector (Gaoge, Shanghai, China). After both sides of the hippocampus were injected, the mice were sutured and then reared for 6 months until they were nine months old. Behavioral tests and MicroPET/CT inspection were conducted, and then the mice were euthanized and their brains were extracted for the following experiment.

### 4.4. Behavioural Tests

#### 4.4.1. MWM Test

The MWM is a test to force rodents to swim and to train them to search for a hidden platform under the water surface and was used to assess learning and memory ability. The apparatus was a white circular pool with a diameter of 100 cm and a height of 50 cm. The pool was imaginarily divided into four equal quadrants, numbered 1–4; the first quadrant was set to be the target quadrant with a cylindrical hidden platform (9 cm diameter, 27 cm height) in the center. A flag, which the mice could use to navigate the maze, was positioned above the pool wall in a constant location during the test. The pool was filled with opaque water mixed with nonfat milk powder, and the water temperature was maintained at 22 ± 1 °C. A video camera above the pool recorded the swimming track of every mouse. All activities of the mice were analyzed by Ethovision XT (Noldus, Wageningen, The Netherlands) monitoring analysis software. The test’s three phases were the visual platform test, hidden platform to start test, and the probe test. At the midpoint of the pool wall of the third quadrant in the first stage, the mice were put in the water to swim with the cue flag. In this stage, the mice should board the visual platform in no more than 60 s and then stand on the platform for 20 s. If the mouse cannot board the platform, it should be guided to the board platform and stand on it for 20 s. The second stage consisted of six consecutive days. In this stage, the platform was submerged 1 cm under the water surface and hidden by opaque milk. Each mouse was put in the water from the midpoint pool wall of any quadrant except the platform quadrant. If the mice explored in the pool and escaped onto the platform for 5 s within 60 s, the total time was recorded as escape latency. If the mouse failed to board the platform in 60 s, it was guided to the platform by the experimenter and stood on it for 15 s. Each mouse was trained three times every day in three different quadrants (one time per quadrant). The time intervals between quadrant training sessions were not less than 30 min. The mice were wiped with a dry towel and dried by heater after each swim. All activities were recorded, including escape latency, distance moved (the distance of every swim in the sixth day), swim speed, etc. During the probe test, the stage platform was removed. The mice were put in the water from the middle of the pool wall of the third quadrant and allowed to swim freely for 60 s. All activities were recorded, including time spent in quadrants, frequency of crossing platform area, swimming trace, etc. All results were analyzed by Ethovision XT (Noldus) monitoring analysis software.

#### 4.4.2. Open Field Test

An open field test was used for tracking autonomous activities, anxiety, and other emotional changes of the animals in a novel open environment. The test apparatus was a white behavioral box 50 cm in length, 50 cm wide, and 40 cm high. It was divided into a border zone, center zone, and intermediate zone. Each mouse was put in the same place within the field and tracked by video camera in five minutes. All activities were analyzed by Ethovision XT (Noldus) monitoring analysis software containing mobile time, immobile time, total distances, frequency of rearing, etc. The apparatus was cleaned with 75% alcohol between every two mice to eliminate odor.

#### 4.4.3. Novel Object Test

The novel object test, an experiment conducted to explore recognition ability due to innate curiosity, prompts rodents to explore novel objects. The apparatus was 50 cm in length, 50 cm wide, and 40 cm high within a quiet environment. The test included three phases; an adaptation stage, familiar stage, and test stage. The first and second day is an adaptation stage in which the mice performed autonomous activities for five minutes each day. The third and fourth day is the familiar stage. Two toy bricks (2 cm × 2 cm) with the same shape, size, and color were put in opposite corners. Each mouse was placed inside for five minutes each day. The fifth day is the probe test stage. One of toy bricks was changed to a different shape and color. Each mouse was placed inside for five minutes. The object probe was defined by the cumulative time of nose within the sniffing zone which was a two cm scope from the object rim. The probe time to new object (TN) and familiar object (TF) of mice in five minutes was recorded by video camera. All behavior results were recorded and analyzed by Ethovision XT (Noldus) monitoring analysis software. The recognition abilities were calculated by the Discrimination Index equation (DI): DI = (TN + TF/TN + TF) × 100%. Between every two mice tests, the apparatus was cleaned with 75% alcohol to eliminate odor.

### 4.5. Glucose Metabolism Detection

The mice were detected for glucose metabolism by MicroPET/CT (INVEON; Siemens, Munich, Germany) when they were nine months old after behavioral tests. They were fasted for more than six hours with free access to water before the inspection. When the examination would be performed, the mice inhaled isoflurane with a 2% anaesthetized dose and a 1.5% maintenance dose. 2-deoxy-2-[^18^F]fluoro-d-glucose (^18^F-FDG) were injected 55 min before the inspection started. The mice were scanned in a prone position. The MicroPET and CT images were captured by the INVEON PET/CT scanner. PET/CT colocalization images were built and data was analyzed by Inveon Research Workspace 4.1 software (Siemens, Knoxville, TN, USA) upon conclusion of the examination. The regions of interest (ROI) containing anterior, middle, and posterior regions of the hippocampus (AH, MH, PH) were selected for analysis. The relative standardized uptake value (SUVr) and % injected dose per gram of ^18^F-FDG (%ID/g) of ROI were calculated and analyzed to evaluate glucose uptake.

### 4.6. Immunohistochemical Staining

Mice brain tissues were fixed with a 10% formalin solution for 48–72 h and then dehydrated and embedded into paraffin and cut into 4 µm coronary sections. Every three hippocampal slices with similar anatomical regions for each mouse were stained with each antibody. The slices were dewaxed, hydrated by gradient alcohol, and then repaired with a sodium citrate buffer and blocked with normal goat serum sealing fluid (ZSGB-BIO, Beijing, China). These were incubated with anti-Aβ17-24 antibody (1:300, 4G8, BioLegend, San Diego, CA, USA), anti-PSD-95 antibody (1:300, Abcam, Cambridge, UK), anti-MAP2 antibody (1:300, abcam), or anti-vimentin antibody (1:100, CST, Framingham, MA, USA) overnight at 4 °C. HRP-labeled anti-rabbit/mouse IgG (ZSGB-BIO) was used to identify antigens, and DAB (ZSGB-BIO) was performed to stain. The slices were collected in images by Olympus cellSens (Tokyo, Japan) software and analyzed by image J (NIH) software (Media Cybernetics Inc., Rockville, MD, USA).

### 4.7. Immunofluorescence Staining

Every three hippocampal slices of the same anatomical locations of each mouse were selected to recognize the corresponding antibody. The sections were dewaxed and hydrated by gradient alcohol. They were repaired with sodium citrate buffer and blocked with normal goat serum sealing fluid (ZSGB-BIO). The samples were incubated with anti-Dbn1 antibody (1:500, Santa, CA, USA), anti-GFAP antibody (1:500, abcam), anti-Cox43antibody (1:300, Abcam), or anti-Syn1 antibody (1:300, Abcam). After washing with PBS buffer (0.01 M), the slices were incubated with FITC/TRITC labeled anti-rabbit/mouse IgG and counterstained by DAPI (ZSGB-BIO). The slices were collected in images by FV10-ASW4.1.2.1 (Olympus, Tokyo, Japan) software and analyzed by image J (NIH) software.

### 4.8. Statistical Analysis

The statistical analyses were performed as one-or two-way ANOVA using SPSS 13.0 for windows (SPSS Inc., Chicago, IL, USA). All results were expressed as means ± SEM. Students’ *t*-test was used for two group analysis. Tukey’s test was used for multiple group analysis. *p* < 0.05 was considered statistically significant.

## 5. Conclusions

In conclusion, this study reveals the impact of down-regulated *Dbn* in the hippocampus of the AD brain. A lack of Dbn results in poor recognitive capability, increases in anxiety, and reductions in glucose utilization. In addition, decreases in PSD-95 and increased vimentin were observed, along with aggravated lesions in AD.

## Figures and Tables

**Table 1:** The primers of ShRNA sequences.

Number	Direction	Oligonucleotide Sequence 5′–3′
1	forward	GATCCGGGCAGTCTATCTTTGGTGACCACTCGAGTGGTCACCAAAGATAGACTGCTTTTTTAGATCTA
	Reverse	AGCTTAGATCTAAAAAAGCAGTCTATCTTTGGTGACCACTCGAGTGGTCACCAAAGATAGACTGCCCG
2	forward	GATCCGGGAGAACCAGAAAGTGATGTATCTCGAGATACATCACTTTCTGGTTCTCTTTTTTAGATCTA
	Reverse	AGCTTAGATCTAAAAAAGAGAACCAGAAAGTGATGTATCTCGAGATACATCACTTTCTGGTTCTCCCG
3	forward	GATCCGGCCAATGGAGAGACCACTCAAACTCGAGTTTGAGTGGTCTCTCCATTGGTTTTTTAGATCTA
	Reverse	AGCTTAGATCTAAAAAACCAATGGAGAGACCACTCAAACTCGAGTTTGAGTGGTCTCTCCATTGGCCG

## Supplementary Materials

Supplementary materials can be found at www.mdpi.com/1422-0067/18/4/800/s1.

## Abbreviations

AD	Alzheimer’s disease
Drebrin	Developmentally regulated brain protein
MWM	Morris water maze
ROI	Regions of interest
FDG	Fluorodeoxyglucose
%ID/g	Percentage injected dose per gram of ^18^F-FDG
SUV	Standardized uptake value
PSD-95	Postsynaptic density-95
Cox43	Connexin43
Syn1	Synapsin1
ANOVA	Analysis of variance

## References

[1] Giese K.P., Aziz W., Kraev I., Stewart M.G. (2015). Generation of multi-innervated dendritic spines as a novel mechanism of long-term memory formation. Neurobiol. Learn. Mem..

[2] Jung G., Kim E.J., Cicvaric A., Sase S., Gröger M., Höger H., Sialana F.J., Berger J., Monje F.J., Lubec G. (2015). Drebrin depletion alters neurotransmitter receptor levels in protein complexes, dendritic spine morphogenesis and memory-related synaptic plasticity in the mouse the hippocampus. J. Neurochem..

[3] Mizui T., Kojima N., Yamazaki H., Katayama M., Hanamura K., Shirao T. (2009). Drebrin E is involved in the regulation of axonal growth through actin-myosin interactions. J. Neurochem..

[4] Mizui T., Sekino Y., Yamazaki H., Ishizuka Y., Takahashi H., Kojima N., Kojima M., Shirao T. (2014). Myosin II ATPase activity mediates the long-term potentiation-induced exodus of stable F-actin bound by drebrin A from dendritic spines. PLoS ONE.

[5] Pollak D.D., Scharl T., Leisch F., Herkner K., Villar S.R., Hoeger H., Lubec G. (2005). Strain-dependent regulation of plasticity-related proteins in the mouse the hippocampus. Behav. Brain Res..

[6] Takizawa H., Hiroi N., Funahashi A. (2012). Mathematical modeling of sustainable synaptogenesis by repetitive stimuli suggests signaling mechanisms in vivo. PLoS ONE.

[7] Tanabe K., Yamazaki H., Inaguma Y., Asada A., Kimura T., Takahashi J., Taoka M., Ohshima T., Furuichi T., Isobe T. (2014). Phosphorylation of drebrin by cyclin-dependent kinase 5 and its role in neuronal migration. PLoS ONE.

[8] Shirao T., González-Billault C. (2013). Actin filaments and microtubules in dendritic spines. J. Neurochem..

[9] Ruzicka J., Kulijewicz-Nawrot M., Rodrigez-Arellano J.J., Jendelova P., Sykova E. (2016). Mesenchymal Stem Cells Preserve Working Memory in the 3xTg-AD Mouse Model of Alzheimer’s Disease. Int. J. Mol. Sci..

[10] Counts S.E., He B., Nadeem M., Wuu J., Scheff S.W., Mufson E.J. (2012). Hippocampal drebrin loss in mild cognitive impairment. Neurodegener. Dis..

[11] Rao J.S., Rapoport S.I., Kim H.W. (2011). Altered neuroinflammatory, arachidonic acid cascade and synaptic markers in postmortem Alzheimer’s disease brain. Transl. Psychiatry.

[12] Liu D.S., Pan X.D., Zhang J., Shen H., Collins N.C., Cole A.M., Koster K.P., Ben Aissa M., Dai X.M., Zhou M. (2015). APOE4 enhances age-dependent decline in cognitive function by down-regulating an NMDA receptor pathway in EFAD-Tg mice. Mol. Neurodegener..

[13] Rusanescu G., Mao J. (2017). Peripheral nerve injury induces adult brain neurogenesis and remodelling. J. Cell. Mol. Med..

[14] Liu Y., Zhou L.J., Wang J., Li D., Ren W.J., Peng J., Wei X., Xu T., Xin W.J., Pang R.P. (2017). TNF-α Differentially regulates synaptic plasticity in the hippocampus and spinal cord by microglia-dependent mechanisms after peripheral nerve injury. J. Neurosci..

[15] Ashok B.S., Ajith T.A., Sivanesan S. (2017). Hypoxia-inducible factors as neuroprotective agent in Alzheimer’s disease. Clin. Exp. Pharmacol. Physiol..

[16] Mascalchi M., Ginestroni A., Bessi V., Toschi N., Padiglioni S., Ciulli S., Tessa C., Giannelli M., Bracco L., Diciotti S. (2013). Regional analysis of the magnetization transfer ratio of the brain in mild Alzheimer disease and amnestic mild cognitive impairment. AJNR Am. J. Neuroradiol..

[17] Winkler E.A., Nishida Y., Sagare A.P., Rege S.V., Bell R.D., Perlmutter D., Sengillo J.D., Hillman S., Kong P., Nelson A.R. (2015). GLUT1 reductions exacerbate Alzheimer’s disease vasculo-neuronal dysfunction and degeneration. Nat. Neurosci..

[18] AbdAlla S., Langer A., Fu X., Quitterer U. (2013). ACE inhibition with captopril retards the development of signs of neurodegeneration in an animal model of Alzheimer’s disease. Int. J. Mol. Sci..

[19] Wang Y., Cella M., Mallinson K., Ulrich J.D., Young K.L., Robinette M.L., Gilfillan S., Krishnan G.M., Sudhakar S., Zinselmeyer B.H. (2015). TREM2 lipid sensing sustains the microglial response in an Alzheimer’s disease model. Cell.

[20] Seo J., Giusti-Rodríguez P., Zhou Y., Rudenko A., Cho S., Ota K.T., Park C., Patzke H., Madabhushi R., Pan L. (2014). Activity-dependent p25 generation regulates synaptic plasticity and Aβ-induced cognitive impairment. Cell.

[21] Clarke L.E., Barres B.A. (2013). Emerging roles of astrocytes in neural circuit development. Nat. Rev. Neurosci..

[22] Helfand B.T., Chou Y.H., Shumaker D.K., Goldman R.D. (2005). Intermediate filament proteins participate in signal transduction. Trends Cell Biol..

[23] Helfand B.T., Mendez M.G., Murthy S.N., Shumaker D.K., Grin B., Mahammad S., Aebi U., Wedig T., Wu Y.I., Hahn K.M., Inagaki M., Herrmann H., Goldman R.D. (2011). Vimentin organization modulates the formation of lamellipodia. Mol. Biol. Cell.

[24] Butkevich E., Hülsmann S., Wenzel D., Shirao T., Duden R., Majoul I. (2004). Drebrin is a novel connexin-43 binding partner that links gap junctions to the submembrane cytoskeleton. Curr. Biol..

[25] Ambrosi C., Ren C., Spagnol G., Cavin G., Cone A., Grintsevich E.E., Sosinsky G.E., Sorgen P.L. (2016). Connexin43 Forms Supramolecular Complexes through Non-Overlapping Binding Sites for Drebrin, Tubulin, and ZO-1. PLoS ONE.

[26] Li H., Spagnol G., Naslavsky N., Caplan S., Sorgen P.L. (2014). TC-PTP directly interacts with connexin43 to regulate gap junction intercellular communication. J. Cell Sci..

[27] Scheff S.W., Price D.A., Ansari M.A., Roberts K.N., Schmitt F.A., Ikonomovic M.D., Mufson E.J. (2015). Synaptic change in the posterior cingulate gyrus in the progression of Alzheimer’s disease. J. Alzheimer’s Dis..

[28] Minter M.R., Moore Z., Zhang M., Brody K.M., Jones N.C., Shultz S.R., Taylor J.M., Crack P.J. (2016). Deletion of the type-1 interferon receptor in APPSWE/PS1ΔE9 mice preserves cognitive function and alters glial phenotype. Acta Neuropathol. Commun..

[29] Jung D., Hwang Y.J., Ryu H., Kano M., Sakimura K., Cho J. (2016). Conditional knockout of Cav2.1 disrupts the accuracy of spatial recognition of CA1 place cells and spatial/contextual recognition behavior. Front. Behav. Neurosci..

[30] Verclytte S., Lopes R., Lenfant P., Rollin A., Semah F., Leclerc X., Pasquier F., Delmaire C. (2016). Cerebral hypoperfusion and hypometabolism detected by arterial spin labeling MRI and FDG-PET in early-onset Alzheimer’s disease. J. Neuroimaging.

[31] Murayama N., Ota K., Kasanuki K., Kondo D., Fujishiro H., Fukase Y., Tagaya H., Sato K., Iseki E. (2016). Cognitive dysfunction in patients with very mild Alzheimer’s disease and amnestic mild cognitive impairment showing hemispheric asymmetries of hypometabolism on ¹⁸F-FDG PET. Int. J. Geriatr. Psychiatry.

[32] Song N., Zhang L., Chen W., Zhu H., Deng W., Han Y., Guo J., Qin C. (2016). Cyanidin 3-*O*-β-glucopyranoside activates peroxisome proliferator-activated receptor-γ and alleviates cognitive impairment in the APP(swe)/PS1(ΔE9) mouse model. Biochim. Biophys. Acta.

[33] Yao X.Q., Jiao S.S., Saadipour K., Zeng F., Wang Q.H., Zhu C., Shen L.L., Zeng G.H., Liang C.R., Wang J. (2015). p75NTR ectodomain is a physiological neuroprotective molecule against amyloid-β toxicity in the brain of Alzheimer’s disease. Mol. Psychiatry.

[34] Cao J., Tang Y., Li Y., Gao K., Shi X., Li Z. (2017). Behavioral changes and the hippocampus glucose metabolism in APP/PS1 transgenic mice via electro-acupuncture at governor vessel acupoints. Front. Aging Neurosci..

[35] Shah K., Desilva S., Abbruscato T. (2012). The role of glucose transporters in brain disease: Diabetes and Alzheimer’s disease. Int. J. Mol. Sci..

[36] Medeiros R., LaFerla F.M. (2013). Astrocytes: Conductors of the Alzheimer disease neuroinflammatory symphony. Exp. Neurol..

[37] Sochocka M., Diniz B.S., Leszek J. (2016). Inflammatory response in the CNS: Friend or foe?. Mol. Neurobiol..

[38] Sajja V.S., Hlavac N., VandeVord P.J. (2016). Role of Glia in Memory Deficits Following Traumatic Brain Injury: Biomarkers of Glia Dysfunction. Front. Integr. Neurosci..

[39] McGeer P.L., McGeer E.G. (2002). Local neuroinflammation and the progression of Alzheimer’s disease. J. Neurovirol..

[40] Li R., Yang L., Lindholm K., Konishi Y., Yue X., Hampel H., Zhang D., Shen Y. (2004). Tumor necrosis factor death receptor signaling cascade is required for amyloid-β protein-induced neuron death. J. Neurosci..

[41] Sultana R., Banks W.A., Butterfield D.A. (2010). Decreased levels of PSD95 and two associated proteins and increased levels of BCl2 and caspase 3 in the hippocampus from subjects with amnestic mild cognitive impairment: Insights into their potential roles for loss of synapses and memory, accumulation of Aβ, and neurodegeneration in a prodromal stage of Alzheimer’s disease. J. Neurosci. Res..

[42] Wang L., Du Y., Wang K., Xu G., Luo S., He G. (2016). Chronic cerebral hypoperfusion induces memory deficits and facilitates Aβ generation in C57BL/6J mice. Exp. Neurol..

[43] Valla J., Gonzalez-Lima F., Reiman E.M. (2008). FDG autoradiography reveals developmental and pathological effects of mutant amyloid in PDAPP transgenic mice. Int. J. Dev. Neurosci..

[44] Wang X., Song Y., Chen L., Zhuang G., Zhang J., Li M., Meng X.F. (2013). Contribution of single-minded 2 to hyperglycaemia-induced neurotoxicity. Neurotoxicology.

[45] Lebkuechner I., Wilhelmsson U., Möllerström E., Pekna M., Pekny M. (2015). Heterogeneity of Notch signaling in astrocytes and the effects of GFAP and vimentin deficiency. J. Neurochem..

[46] Jiang S.X., Slinn J., Aylsworth A., Hou S.T. (2012). Vimentin participates in microglia activation and neurotoxicity in cerebral ischemia. J. Neurochem..

[47] George Paxinos K., Franklin B.J. (2001). The Mouse Brain in Stereotaxic Coordinates.

