# Supplementary material for: Down-Regulated Drebrin Aggravates Cognitive Impairments in a Mouse Model of Alzheimer’s Disease
Source: Int J Mol Sci. 2017 Apr 11;18(4):800. doi: 10.3390/ijms18040800 (PMC5412384; doi:10.3390/ijms18040800)
Supplement: Supplementary file 1 [file ijms-18-00800-s001.pdf]

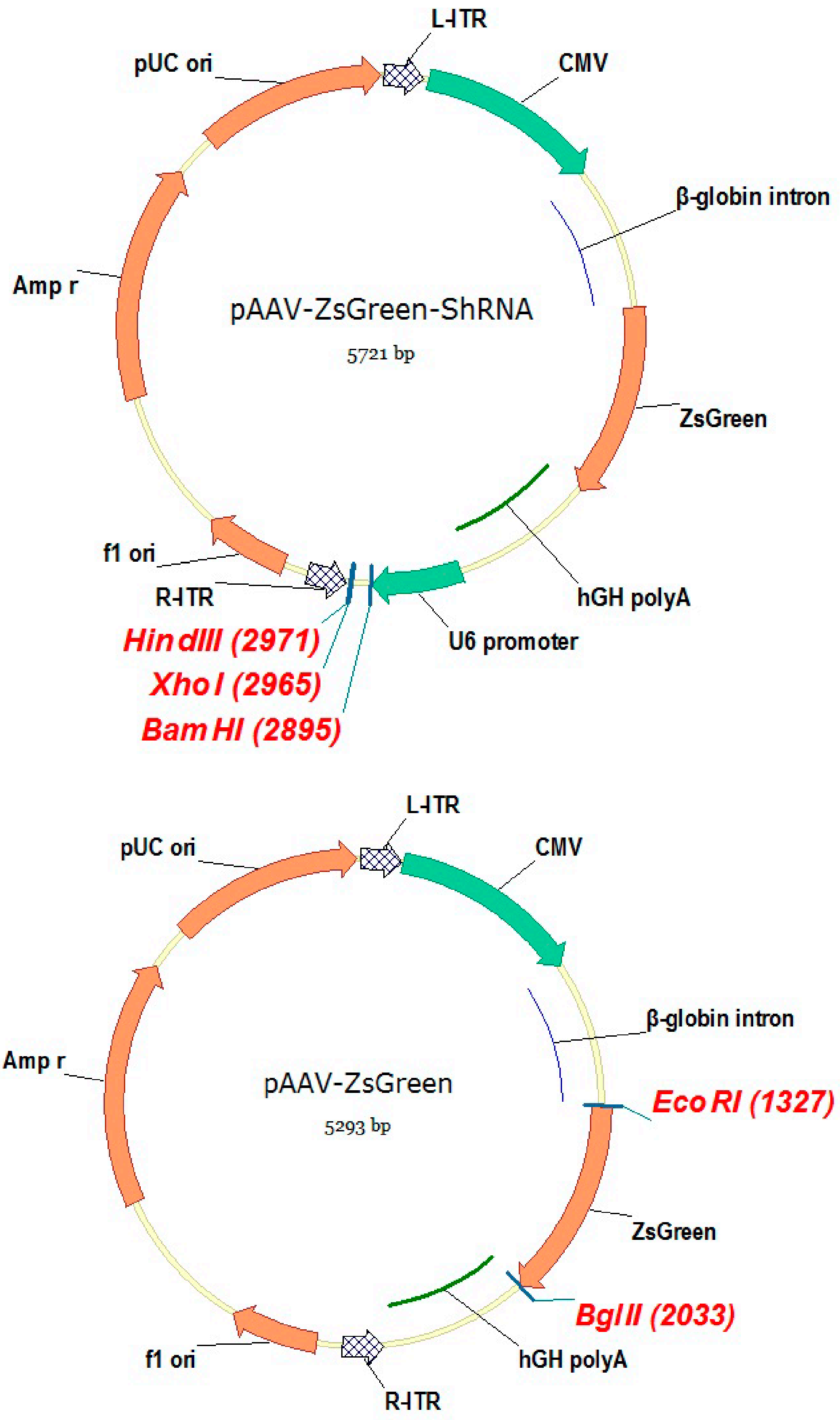

Figure S1. Basic structure of pAAV-ShRNA plasmid contains enzyme identification and Control plasmid.
